# Impact of biofertilizers from goat and sheep manure on rhizospheric microbial community of *Cenchrus ciliaris*

**DOI:** 10.1007/s42770-026-01938-4

**Published:** 2026-05-11

**Authors:** Jennifer Figueiredo da Silva Oliveira, Gisele Veneroni Gouveia, João José de Simoni Gouveia, Luciana Correia de Almeida Regitano, Wilson Malago-Jr, Danillo Sales Rosa, Mário Adriano Ávila Queiroz, Mateus Matiuzzi da Costa, Adriana Mayumi Yano-Melo

**Affiliations:** 1 Senhor do Bonfim, Instituto Federal Baiano, Bahia, 48970-000 Brazil; 2 Universidade Federal Do Vale Do São Francisco, Campus Ciências Agrárias, Petrolina, Pernambuco 56300-990 Brazil; 3 Embrapa Pecuária Sudeste, São Carlos, São Paulo, 13560-970 Brazil; 4 Universidade Federal Rural de Pernambuco, Recife, Pernambuco 52171- 900 Brazil

**Keywords:** Bioinformatics, Buffel-grass, Microbial diversity, Organic matter, OTU, 16S r RNA

## Abstract

Use of biofertilizers has been increasing to enhance plant growth, yet less attention has been given to their effects on the microbial community and functional composition of the rhizosphere. In this study, biofertilizers produced from sheep (BO) and goat (BC) manure were tested at concentrations ranging from 0 to 10% of soil volume over two vegetative cycles of *Cenchrus ciliaris*. As expected, biofertilizer application increased CFU counts of coliforms and microbial biomass carbon (MBC). However, the effects on rhizospheric soil biological and chemical properties varied depending on the biofertilizer source. BO increased organic matter (OM) content, whereas BC increased some nutrients, electrical conductivity and the number of total fungi while reducing the number of glomerospores and mycorrhizal colonization. Higher relative abundance of OTUs was found with pure BO, indicating that its application increased microbial diversity and functional composition compared to BC. Moreover, biofertilizer application in the second vegetative cycle suggested that its continuous use may impact the rhizospheric microbial community. These results suggest that the source of biofertilizer and the application of parsimonious doses may prevent the loss of microbial diversity and maintain rhizospheric soil functional capacity.

**Supplementary Information:**

The online version contains supplementary material available at 10.1007/s42770-026-01938-4.

## Introduction

The microbiological parameters of soil have increasingly shown potential as early and sensitive indicators of soil stress, and it can be inferred that the reduced enzymatic activity, biomass, and microbial diversity are related to the lower productive capacity due to the roles played by microorganisms [1].

Bacterial soil communities are responsible for numerous processes and functions, presenting a fundamental role in nutrient dynamics in different ecosystems [2]. Thus, many researchers have been demonstrating the intrinsic relationship between the increase in microbial biomass carbon (MBC) and greater availability of organic substrates in soil [3]. These organic substrates are relevant for agricultural production, where the degradation of organic matter is accelerated and the effect on the physical, chemical, and biological properties of the soil are greatly intensified [4].

Fungal communities are also important components of soil microbiota, especially in the rhizosphere where interactions with plant roots occur. Among them, arbuscular mycorrhizal fungi (AMF) establish symbiotic relationships with plants, improving nutrient uptake and contributing to soil structure and nutrient cycling. Indicators such as mycorrhizal colonization and the number of glomerospores are commonly used to evaluate AMF activity in soils. However, these organisms can be influenced by soil management practices and nutrient availability, including the application of organic amendments [5, 6].

Manure-based biofertilizers generated in animal production are important sources of organic matter for restoring soil quality, and they act as a positive factor in sustainable land management practices [7].

In this context, in regions where goat and sheep farming is widespread, such as the semiarid regions of Brazil, the manure of these animals can serve as a viable alternative source of biofertilizer. Livestock production in Brazil, based mainly on pastures, plays an important role in animal production and has encouraged investment in goat and sheep farming, especially in the northeast region, which has a semiarid climate and is home to more than half of the country’s goat and sheep herds [8]. In practice, buffel-grass (*Cenchrus ciliaris* L.) is the main forage grass cultivated in important ruminant-producing regions of the Brazilian semiarid region, as it is forage species well adapted to semiarid conditions, known for to its high water use efficiency, tolerating periods of drought and favoring the availability of quality forage, with high nutritional value, good digestibility of dry matter and crude protein, and good palatability [9]. It is an invasive plant whose establishment strategy is allelopathy through root exudates, releasing compounds that can alter the composition of the microbial community of the rhizospheric soil, recruiting micro-organisms involved in metabolising the allelochemicals produced [10].

Researches that aim to evaluate the production of biofertilizers with goat and sheep manure and the understanding of the fungal and bacterial structure and prediction of the functions of the rhizospheric soil of *C. ciliaris* with the addition of this effluent may allow an understanding of the ecology of this use, which may serve as an indicator of the effect of different anthropic activities [11].

It is worth highlighting that wastes applied inappropriately may have a potential for pollution or contamination. In this perspective, the origin and doses of applied biofertilizers are important for the economic feasibility and sustainability of the process of waste use due to the risks of environmental contamination, especially by pathogenic microorganisms [12].

Given the above, it was hypothesized that the application of biofertilizers produced from goat and sheep manure modifies the chemical and biological properties of the rhizosphere soil of *C. ciliaris*, influencing the structure and functional potential of the microbial community, and that these effects vary according to the source of the biofertilizer and the doses applied in successive vegetative cycles. The main microorganisms found in manure are from the Proteobacteria, Firmicutes, Spirochaetae and Actinobacteria phyla. Notably, groups from the families Enterobacteriaceae, Pseudomonadaceae, Xanthomonadaceae are abundant, presenting bacteria with potential risks to soil, plants, animals and humans. In addition, species of the Clostridia class, commonly found in the intestines of animals, also increase in the soil after the application of manure, bacteria of an anaerobic and spore-forming nature that persist even after anaerobic digestion. It is therefore appropriate to assess the abundance and composition of the main groups of bacteria to ensure that plant production is not contaminated [13].

Therefore, the purpose of the present study was to evaluate the effect of biofertilizers produced from sheep (BO) and goat (BC) manure on the microbial community and functional composition in the rhizosphere of *C. ciliaris* for two vegetative cycles.

## Materials and methods

### Place of experiment

The experiment was carried out under greenhouse conditions, with an average air temperature of approximately 27 °C and relative humidity of 77%, according to the data from the weather station of Universidade Federal do Vale do São Francisco (http://labmet.univasf.Edu.br/joomla/index.php/o-labmet). The experiment was performed in a greenhouse, between January and April 2014.

### Greenhouse experiment

The experiment was conducted in a greenhouse in Petrolina, Pernambuco, Brazil. The daily mean temperature ranged from 27.5 to 28.5 °C, with maximum temperatures ranging from 32 to 34 °C and minimum temperatures ranging from 23 °C. These conditions reflect the region’s typical semi-arid climate, characterized by high temperatures and low relative humidity.

Seeds of *Cenchrus ciliaris* cv. Biloela were provided by Embrapa Semiárido, Brazil. The proceeding used to treat dormancy breaking in seeds of *C. ciliaris* cv. Biloela followed the recommendation of the Ministry of Agriculture, Livestock and Food Supply (MAPA – Ministério da Agricultura, Pecuária e Abastecimento, Brazil) [14]. They were exposed to a temperature of 5 °C for a period of 7 days, followed by 15 h in a water bath at 53 °C, and subsequent addition of potassium nitrate solution at 2%. After that, seeds were washed with distilled water and transferred to pots. In this study, the seeds of *C. ciliaris* were sown without prior disinfestation, in accordance with the common practice reported in the literature [15, 16]. No negative effects on germination have been observed under these conditions.

To obtain seedlings, 50 seeds of *C. ciliaris* were placed in pots (5 L) at approximately 1.0 cm depth, followed by thinning to maintain only three plants per experimental unit. After seven days, the pots were fertigated with 0, 2.5, 5.0, 7.5, and 10% of a biofertilizer for the soil volume. These doses were fractionated in four applications (days 1, 7, 14, and 21) and repeated in the second vegetative cycle. The end of each cycle occurred when the plants had 30% of inflorescence (i.e., after approximately 25 days), which represents a condition of equilibrium between dry matter production and nutritional quality. Therefore, after the first vegetative cycle, the grass was cut at 10 cm from the soil level in each experimental unit, and the second vegetative cycle followed the same biofertilizer application schedule.

The pots were irrigated daily with water until they reached the humidity corresponding to 80% of the soil field capacity, which was previously determined through the gravimetric method.

The initial rhizospheric soil used, collected before the application of biofertilizers, was classified as typical dystrophic Yellow Argisol, with a sandy/medium texture, and presenting the following chemical characteristics: 0.04 g kg^− 1^ P, 0.06 g kg^− 1^ K, 0.68 g kg^− 1^ Ca, 012 g kg^− 1^ Mg, 3.28 mg kg^− 1^ Cu, 14.46 mg kg^− 1^ Fe, 11.94 mg kg^− 1^ Mn, 4.40 mg kg^− 1^ Zn, 2.5 g kg^− 1^ OM, and pH of 8.59.

Rhizospheric soil samples from each experimental unit were collected in three periods: (a) before the application of the biofertilizers, (b) at the end of the first vegetative cycle, and (c) at the end of the second vegetative cycle.

### Production of biofertilizers

Two biofertilizers (BO and BC) were produced from the anaerobic biodigestion of manure from mixed-breed animals, whose animals were kept in individual bay and consumed a diet composed of elephant grass silage (*Pennisetum purpureum* Schumach.), a concentrate of corn, soybean meal, and mineral blend (50% bulk and 50% concentrate). After 30 days of adaptation to the diet, manure were collected and the substrates were prepared for supply to the biodigester bench with a capacity of 10 L, using 750 g of feces, previously dried at 55 °C for 72 h, in a circulation and air renewal oven, 550 g of fresh inoculum was produced from fresh manure, bovine rumen liquid, and 7.8 L of peptone water (2.05%), for dilution, with a final total solid content of approximately 4%. The biodigesters remained active for 70 days in order to reduce or eliminate pathogenic microbial agents from feces, following the daily production of gases. The pure biofertilizers produced were stored in a freezer with a temperature between − 10 to −15 °C for a period of 20 days until further [17]. Bovine rumen fluid was included as part of the inoculum to provide an active anaerobic microbial consortium necessary to initiate the fermentation process and promote efficient degradation of organic matter during biodigestion and to avoid failure or delay in fermentation. Without inoculation, the process can have a long lag phase, incomplete fermentation, and low biogas production.

### Experimental design

The experimental design used in each vegetative cycle was completely randomized in a factorial arrangement with two types of biofertilizers (BO and BC) and five doses of each biofertilizer (i.e., 0, 2.5, 5.0, 7.5, and 10%), with four replicates for each treatment.

### Chemical characterization of the rhizospheric soil

The rhizospheric soil samples from each experimental unit were subjected to chemical analysis, and the content of nitrogen (N), phosphorus (P), and potassium (K) for the two vegetative cycles were calculated [18]. Organic matter (OM) contents, electrical conductivity (EC), and cation exchange capability (CEC) of the rhizospheric soil were analyzed only at the end of the second cycle [19].

### Microbial characterization of the samples collected by the culture method

To quantify rhizospheric soil bacteria and total fungi, the technique of serial dilution and plating in media specific for each bacterial group was used: Violet Red Bile (VRB) to enumerate the enterobacteria (ENT), Reinforced Clostridial Medium (RCM) to detect the presence of *Clostridium* spp. (CL) and Xylose-Lysine-Deoxycholate (XLD) Agar, Hektoen Enteric (HE) Agar, and Bismuth Sulfite (BS) Agar for the presence of *Salmonella* spp. and Potato Dextrose Agar (PDA) with addition of chloramphenicol for fungal counting.

### Quantification of Arbuscular Mycorrhizal Fungi

To evaluate the effect of the biofertilizer on the arbuscular mycorrhizal fungi, mycorrhizal colonization was quantified from root separation, clarification, and staining by [9] and the percentage of colonization by the intersection of the quadrants [10]. Mycorrhizal colonization was quantified only in the second vegetative cycle when the plants were harvested. The number of glomerospores was obtained after the extraction of the spores of the rhizospheric soil, by the methods of wet sieving and centrifugation in water and sucrose [6], being the counting done to the stereomicroscope.

### Determination of the MBC of the samples

MBC is the life fraction of the organic matter responsible for biochemical and biological processes, significantly altered by the conditions imposed by the environment [20]. To evaluate the microbial activity of the rhizospheric soil, the MBC was estimated by fumigation with ethanol-free chloroform in 20 g rhizospheric soil, followed by carbon extraction with 50 mL potassium sulfate (0.5 M) and oxidation with 2 mL potassium dichromate (0.66 mM) in a medium containing 10 mL concentrated sulfuric acid and 5 mL concentrated phosphoric acid. Carbon was quantified through titration with ammoniac ferrous sulfate (0.033 N), using 1% diphenylamine as indicator. The values were expressed in µg C g^− 1^ dry rhizospheric soil.

### Statistical analysis

The experiment was conducted in a completely randomized experimental design in a factorial arrangement (2 × 5), two types of biofertilizers (sheep and goat) x five doses of biofertilizers (0, 2.5; 5.0; 7.5 and 10.0), in four replicates. All response variables were analyzed separately in two distinct consecutive vegetative cycles.

Colonization data were expressed as a percentage, and glomerospores data were subjected to the Shapiro-Wilk test to verify the normal distribution of residues and homogeneity of variances by Levene. Residues that did not present a normal distribution were transformed by the root (NG + 1). Coliform and fungal count data were also transformed by (log10(x + 1)).

Analysis of variance was performed by the GLM procedure compawred by orthogonal contrasts (linear, quadratic and deviation from the quadratic) with a significance level of 5%. After the contrast analyses, when significant, the parameters of the regression equations were determined by PROC REG. It is worth noting that preliminary analyses considered both LSD and Duncan post hoc tests for mean comparison; however, given the graded doses of biofertilizers, regression models were deemed more robust and biologically meaningful for describing dose–response relationships.

The model included the treatment effect (types of biofertilizers), doses and interaction effect. The results were expressed as the mean ± standard error of the mean (s.e.) and differences were considered significant when *p* < 0.05. All tests were performed using the SAS 9.2 statistical package.

### Bacterial characterization of the samples through molecular techniques

Samples of the two pure biofertilizers and a pool of replicates of each treatment at the end of each vegetative cycle, totaling 22 rhizospheric soil samples, were subjected to total DNA extraction using a specific kit (the Soil DNA Isolation kit NorgenBiotek Corporation). Thereafter, the DNA samples were used in polymerase chain reaction (PCR) with the following universal primers for prokaryotes for region V4 of the 16S rRNA gene, with the PCR primers being added adapters (according to the recommendations described at: http://www.illumina.com/systems/miseq/applications.ilmn), so that the amplicons could be sequenced: **520F**: 5’TCGTCGGCAGCGTCAGATGTGTATAAGAGACAGAYTGGGYDTAAAGNG 3’ and **802R**: 5’GTCTCGTGGGCTCGGAGATGTGTATAAGAGACAGTACNVGGGTATCTAATCC 3’ [21]. Polymerase chain reaction (PCR) was performed in a final volume of 50 µL, containing 21.7 µL of ultrapure water, 5 µL of 10× PCR buffer, 2 µL of MgCl₂ (25 mM), 2.5 µL of dNTP mix (10 mM), 3 µL of each primer, 0.8 µL of Taq DNA polymerase (5 U/µL), and 7 µL of template DNA.

Thermal cycling was carried out under the following conditions: initial denaturation at 95 °C for 5 min, followed by 40 cycles of denaturation at 95 °C for 1 min, annealing at 53.1 °C for 1 min, and extension at 72 °C for 2 min. A final extension step was performed at 72 °C for 10 min.

The PCR products were purified using magnetic beads (Agencourt AMPure XP) and quantified through spectrophotometry. The purified product underwent a second PCR to connect the Index adapters, using the Illumina Nextera XT Index kit and purified again with magnetic beads. The samples were placed in a single pool, which was subjected to agarose gel electrophoresis, followed by purification (Zymoclean Gel DNA Recovery KIT). The purified pooled sample was quantified (absolute quantification) by real-time PCR using the KAPA Library Quantification Kit. Subsequently, 10 pM DNA was sequenced on the MiSeq (Illuminaa^®^) equipment using MiSeq Reagent Kit V2 250 cycles of 15 gigabytes.

#### Analysis of 16 S rRNA gene sequences

The raw sequences obtained from the MiSeq ^®^ platform (Illumina) were subjected to analysis for quality control using the FastQC software (https://www.bioinformatics.babraham.ac.uk/projects/fastqc/).

Using the SeqClean software (https://sourceforge.net/projects/seqclean/files/), the sequences were trimmed, with removal of contaminants and sequences of low quality, applying a minimum Phred quality score of 30 (corresponding to a maximum error rate of 0.1%) and retaining sequences with a minimum length of 150 bp. Potential contaminants were removed using a reference file containing known artifacts and adaptors.

The screened sequences were analyzed with the Mothur software [22], as described online (http://www.mothur.org/wiki/Download_mothur). Paired-end reads were merged into contigs using the Needleman-Wunsch alignment algorithm. Sequences containing ambiguous bases or exceeding a maximum length of 275 bp were excluded. Unique sequences were identified to reduce redundancy prior to alignment. Sequence alignment was conducted against the SILVA bacterial reference database (release compatible with Mothur v1.33.3), allowing sequence flipping when necessary to optimize orientation. Alignment quality was refined through the removal of poorly aligned regions and homopolymers exceeding 8 consecutive identical nucleotides. Subsequently, pre-clustering was performed allowing up to 2 nucleotide differences to further minimize sequencing errors. Chimeric sequences were identified and removed using the UCHIME algorithm in de novo mode, referencing the SILVA database. Taxonomic classification was then conducted with the RDP classifier (Trainset 9), applying an 80% bootstrap confidence threshold. Sequences classified as chloroplast, mitochondrial, archaeal, eukaryotic, or unknown origin were excluded from downstream analyses using the remove.lineage command in Mothur. Operational Taxonomic Units (OTUs) were delineated at a 97% similarity threshold using the average neighbor clustering algorithm.

Alpha diversity analyses were performed with the aim of generating exploratory estimates of microbial richness and diversity through the calculation of rarefaction curves and the Chao, ACE, Shannon, and Inverse Simpson indices. Theta-YC dissimilarity was estimated based on a distance matrix (cutoff 0.01) and visualized using Principal Coordinates Analysis (PCoA) to describe beta diversity trends among the biofertilizer and rhizospheric soil samples, and are presented in the Supplementary Material.

The functional content of the sequences was predicted using the PICRUSt software, designed to predict metabarcoding functional content from marker gene (e.g., 16 S rRNA) surveys and full genomes. For this analysis, a closed reference OTU selection strategy was used using Qiime software [23], by removing all OTUs that do not match the GreenGenes 13_5 reference sequences with 97% similarity. The OTUs were normalized by dividing their abundances by known or predicted number of copies with subsequent metabarcoding functional classifications based on the KEGG Orthology (Kos) database.

We emphasize that, due to the absence of biological replicates, the diversity and functional content results are purely descriptive and do not allow for statistical inference.

These sequence data have been submitted to the GenBank databases under accession number KBZG00000000.

## Results

### Chemical attributes of rhizospheric soil treated with different biofertilizer doses

Soil OM content increased significantly with increasing doses of BO. The content was the maximum at the highest dose tested. These effects were estimated according to the following regression equation: ŷ_BO_ = 2.19 + 0.33 X (R^2^ = 0.60; Fig. S1a). However, EC showed a significant interaction effect between biofertilizer type and dose. A positive linear increase in OM content was found dose improvement of both the biofertilizers. The highest values were obtained by BC application (Fig. S1b). No difference in CEC was found between the two biofertilizers, but an increasing linear response was found in response to an improvement in the doses (Fig. S1c).

**Fig. 1:**
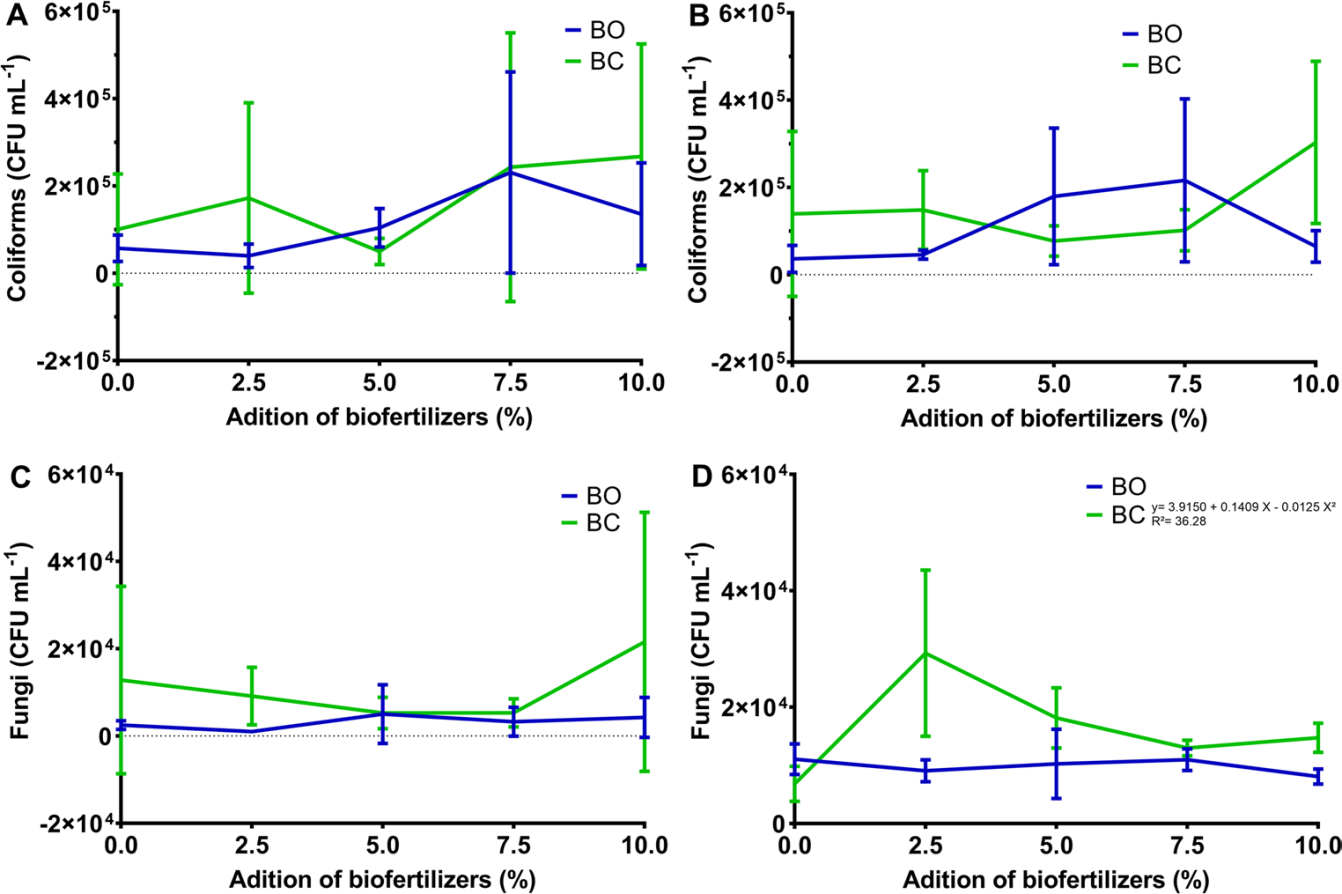
Effect of the application of biofertilizers, produced by anaerobic biodigestion of sheep (BO) and goat (BC) manure, at different doses (0, 2.5, 5.0, 7.5, and 10% of rhizospheric soil volume), on the CFU number of total coliforms (**A** and **B**) and fungi (**C** and **D**) of the rhizospheric soil in two vegetative cycles of *C. ciliaris*. A and C refer to cycle 1. B and D refer to cycle 2

Application of BC resulted in higher levels of N, P, and K, thus contributing to an improvement in rhizospheric. In the first vegetative cycle, BC when compared to BO had a significant effect on N content (ŷ_BC_ = 1.25 + 1.35 X − 0.03 × ^2^; R^2^ = 0.62) (Fig. S1) and P content (ŷ_BC_ = 1.64 + 0.04 X + 0.015 × ^2^; R^2^ = 0.87) (Fig. 1e) as compared with that of BO. Already in the second cycle an interaction effect was found between the biofertilizers for N content (ŷ_BC_ = 9.81 + 0.26 X + 0.03 × ^2^, R^2^ = 0.77; ŷ_BO_ = 9.48 + 0.35 X, R^2^ = 0.88) (Fig. S1f) and K content (ŷ_BC_ = 20.17 + 6.0 X − 0.57 × ^2^, R^2^ = 0.87; ŷ_BO_ = 21.37 + 2.26 X, R^2^ = 0.86) (Fig. S1g). BC provided higher levels of these macronutrients to the rhizospheric soil. For all analyses, *p* < 0.05 was used.

### Bacterial and fungal characterization of biofertilizers and rhizospheric soil by culture method

Before the addition of biofertilizers, the rhizospheric soil contained total coliforms of 268 × 10³ CFU mL⁻¹ and total fungi of 2 × 10³ CFU mL⁻¹. The biofertilizers prior to fermentation contained lower counts of total coliforms, with BO at 81.7 × 10³ CFU mL⁻¹ and BC at 59.9 × 10³ CFU mL⁻¹, and total fungi of 2 × 10³ CFU mL⁻¹ and 1.3 × 10³ CFU mL⁻¹, respectively. After 70 days of fermentation in biodigesters, coliform counts decreased to 5 × 10³ CFU mL⁻¹ in BO and 1.5 × 10³ CFU mL⁻¹ in BC, while fungal counts increased slightly to 5.2 × 10³ CFU mL⁻¹ in BO and 6.2 × 10³ CFU mL⁻¹ in BC.

For samples collected at the end of the first cycle of *C. ciliaris*, for the number of total coliforms of the rhizospheric soil samples, no significant difference was found between the types of biofertilizers (BO and BC) used or doses applied (Fig. 1A and B). There was an increase in the number of total coliforms with the application of increasing doses of biofertilizers, with doses of 7.5–10% providing an average increase of 240% as compared with that in the control (without biofertilizer) (Fig. 1A). For the rhizospheric soil samples collected at the end of the second vegetative cycle, there was an interaction effect between the types of biofertilizers and the doses applied. A significant increase in the number of total coliforms was found using BC at a dose of 10%, thereby increasing the number of CFU by 671% as compared with that in the control (Fig. 1B). However, the application of BO increased the number of total coliforms up to the dose of 7.5%, followed by a reduction at a 10% BO application (Fig. 1B).

*Clostridium* spp. was not found in any of the rhizospheric soil and biofertilizer samples by the cultivation method. *Salmonella* spp. were already present in the rhizospheric soil before biofertilizer application, and they were absent from the biofertilizers used in the present study.

There was a growing increase in the amount of CFU of total fungi, when compared to the amount found in the initial soil collection (before the application of biofertilizers - average 2.13 × 10^3^ CFU mL^− 1^) and after the second vegetative cycle, for all doses and biofertilizers.

Regarding the number of total fungi, there was an interaction effect between the doses and the types of biofertilizers in the first vegetative cycle, there being a statistical difference (*P* = 0.0015) between BC (A = mean) and BO (B = mean), highlighting that BC promoted an increase in the amount of CFU of total fungi with the increase in the doses (Fig. 1C). Consequently, there was an increase of 200 and 235%, respectively for 2.5 and 10% of BC, of these microorganisms in the rhizospheric soil concerning the control. In the second cycle, there was a significant effect of the doses (*P* = 0.0051), the type of biofertilizer used (*P* = 0.0002) and interaction (*P* = 0.0001; ŷ_BC_ = 3.9150 + 0.1409 X – 0.0125 X²; R²= 36.28; ŷ_BO_ = mean) (Fig. 1D). The dose of 2.5% of BC led to a maximum peak of total fungi (29.25 × 10^3^ CFU mL^− 1^), reducing the amount with increasing doses.

In both cycles, BO presented a lower number of total fungi in the rhizospheric soil than BC, and in the second cycle, all doses of BC applied increased the amount of CFU concerning the control (Fig. 1C and D).

Regarding the MBC, the increase in the doses of biofertilizers promoted a linear increase in the means of MBC in the first vegetative cycle (Fig. S2a). However, at the end of the second cycle, an interaction among the doses and the types of biofertilizers was found, with a significant effect only for BO. In this case, the addition of BO reduced linearly the values of MBC (Fig. S2b).

### Arbuscular Mycorrhizal Fungi and number of glomerospores in rhizospheric soil with application of biofertilizers

**Fig. 2:**
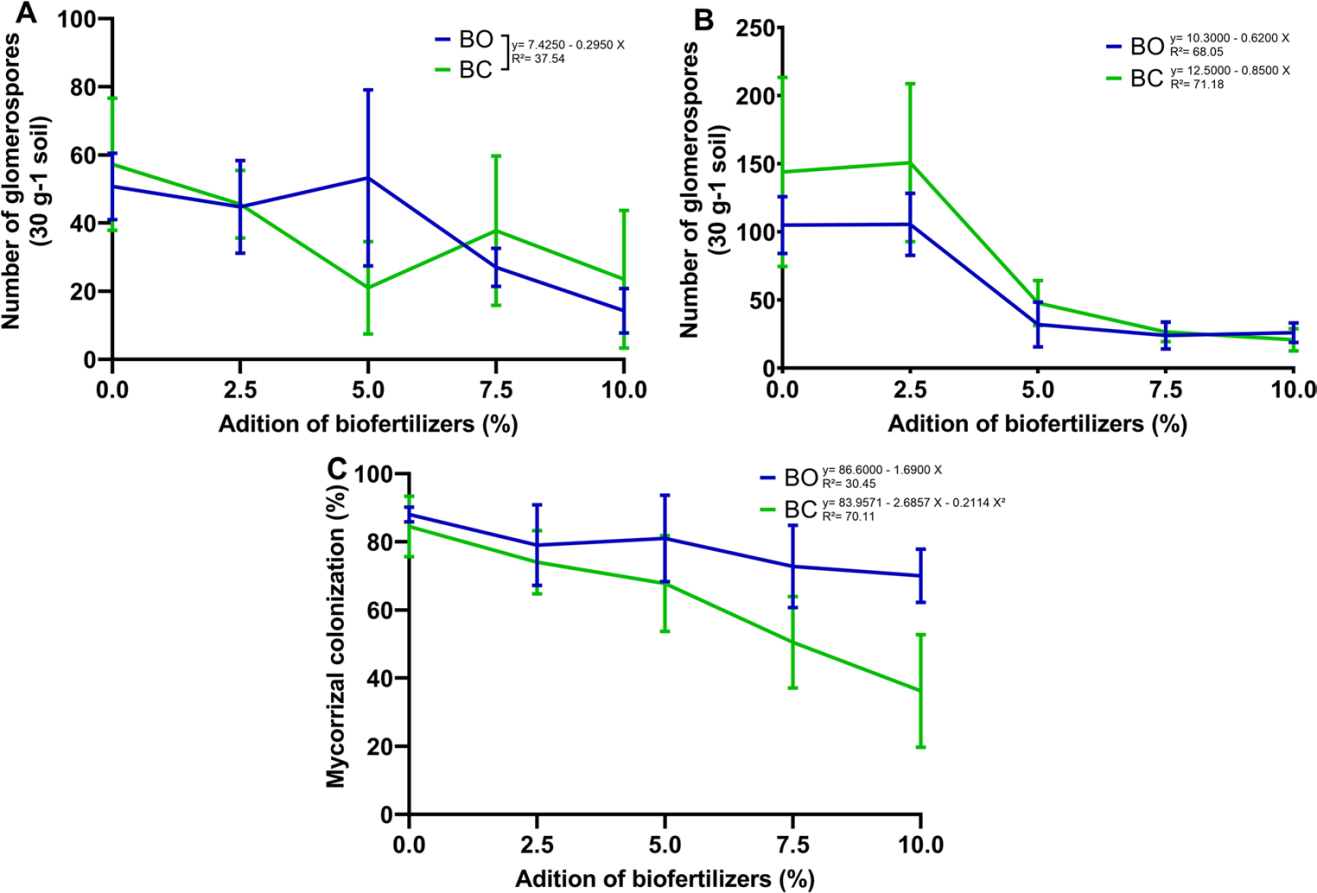
Effect of applying biofertilizers from sheep (BO) and goat (BC) manure at different doses (0, 2.5, 5.0, 7.5, and 10% of soil volume) on the number of glomerospores (**A** and **B**) and mycorrhizal colonization (**C**) of the rhizospheric soil in two vegetative cycles of *C. ciliaris*

There was a significant effect between the biofertilizer doses in the first cycle for the number of glomerospores (*P* = 0.0007; ŷ = 7.4250 − 0.2950 X²; R²= 37.54), with a decrease in the number of glomerospores with the increase in the doses of biofertilizers (Fig. 2a). In the second cycle, there was an effect of the type of biofertilizer (*P* = 0.0271; ŷ_BC_ = 12.5000–0.8500 X; R²= 71.18; ŷ_BO_ = 10.3000–0.6200 X; R²=68.05) and the applied doses (*P* < 0.0001), being observed that from 5.0% of biofertilizer, regardless of the type, the number of glomerospores decreased markedly (Fig. 2b).

Similarly, mycorrhizal colonization was differentially affected by the types (*P* = 0.0002; ŷBC = 83.9571–2.6857 X − 0.2114 X²; R² = 70.11; ŷBO = 86.6000–1.6900 X; R² = 30.45) and doses (*P* < 0.0001) of biofertilizers. Increasing the doses of both biofertilizers affected mycorrhizal colonization. For dose 7.5% of BC, there was a reduction in this variable (Fig. 2c). In the same way, only from the dose 7.5% was observed difference between the types of biofertilizers.

### Bacterial characterization of biofertilizers and rhizospheric soil using molecular techniques

Because sequencing analyses were conducted using pooled samples without biological replicates, the diversity indices and ordination analyses should be interpreted only as descriptive ecological trends rather than inferential comparisons.

A total of 610,854 raw 16 S rRNA gene amplicon sequences targeting the V4 region were obtained from 22 rhizospheric soil samples representing different biofertilizer types and application doses (0, 2.5, 5, 7.5, and 10% of soil volume). After quality filtering and trimming, 340,172 high-quality sequences were retained for downstream analysis. The filtered sequences were clustered into operational taxonomic units (OTUs) at 97% sequence similarity. The number of observed OTUs per sample ranged from 1,675 to 3,440, indicating substantial bacterial diversity across the rhizosphere samples (Table S1).

Figure S3 (Supplementary Material) shows a significant difference between the number of OTUs of the pure biofertilizers and the rhizospheric soil samples treated with increasing doses of the biofertilizers used in the present study. The pure BO tended to present a higher number of OTUs than that BC. Similarly, rhizospheric soil samples receiving BO showed a tendency toward higher OTU richness compared with those receiving BC for most of the tested doses. Moreover, a tendency toward lower OTU numbers was observed from the first vegetative cycle to the second, within each dose tested, except for the rhizospheric soil treated with BO at 5% of the soil volume, which increased the number of OTUs (Fig. S3).

**Fig. 3:**
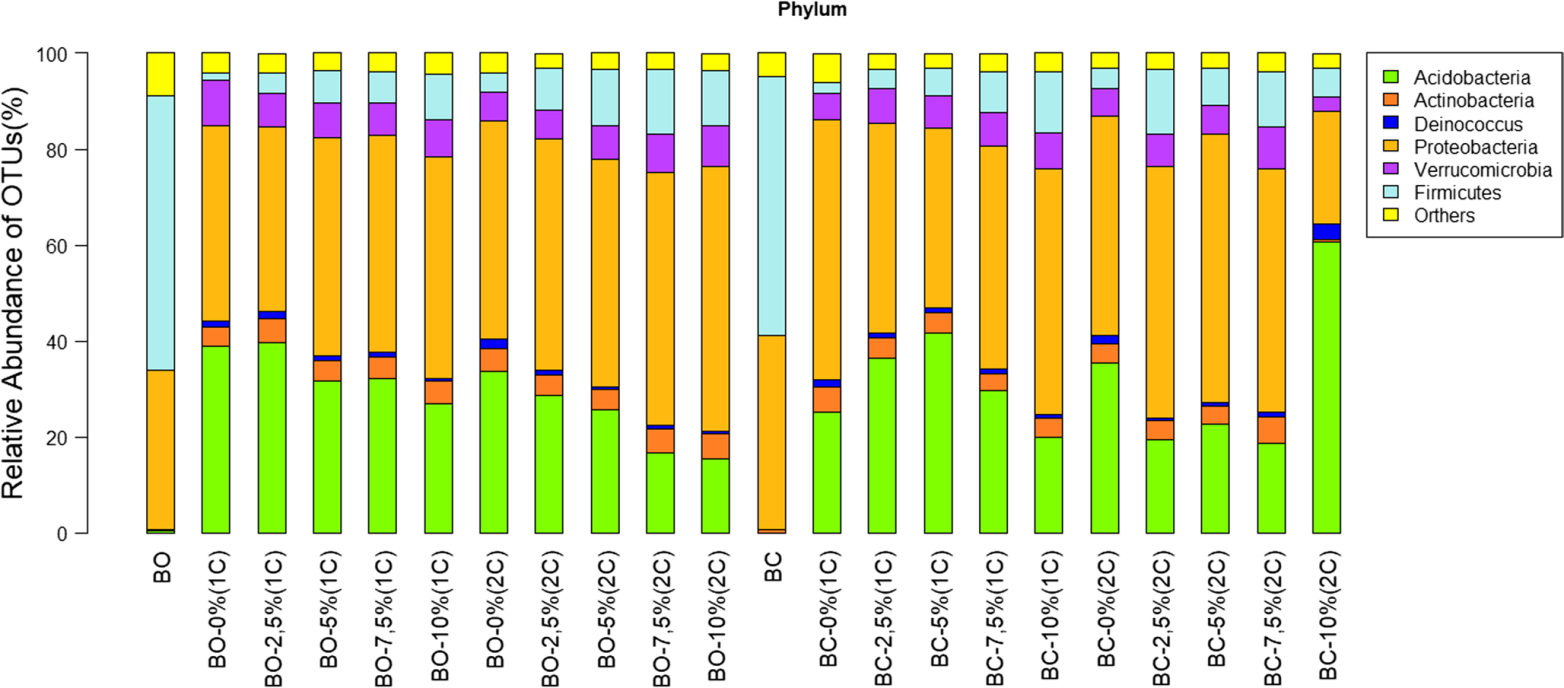
Relative abundance of OTUs (Acidobacteria, Actinobacteria, Deinococcus, Proteobacteria, Verrucomicrobia, and Firmicutes) present in biofertilizers from sheep (BO) and goat (BC) manure and rhizospheric soils treated with increasing doses of these biofertilizers (0, 2.5, 7.5, and 10% of the soil volume) in two vegetative cycles of *C. ciliaris* (1 C: first cycle; 2 C: second cycle)

Considering the total sampling (rhizospheric soils with pure treatments and biofertilizers), the 161,420 OTUs found were attributed to 21 phyla, 51 classes, 85 orders, 167 families, and 364 genera, and 39,845 OTUs were not identified. Considering UTOs attributed to phyla, the most abundant phyla (above 1% of total OTUs) were Proteobacteria (46.06%), Acidobacteria (27.92%), Firmicutes (10.3%), Verrucomicrobia (6.76%), Actinobacteria (4.21%) and Deinococcus (1.02%) (Fig. 3).

Considering only OTUs found in pure biofertilizers, it was found that the phyla Acidobacteria, Verrucomicrobia, Actinobacteria, and Deinococcus were absent or in low abundance of OTUs (< 1%). When biofertilizers were added to the soil, an increase in the relative abundance of these groups was observed, particularly at BO doses of 2.5 and 10% of soil volume in the two vegetative cycles (Fig. 3).

Moreover, 57.16% (BO) and 53.83% (BC) of the OTUs of the phylum Firmicutes and 33.06% (BO) and 40.06% (BC) of the phylum Proteobacteria were present in the pure biofertilizers (Fig. 3). The addition of BO doses to the rhizospheric soil increased the populations of the other phyla, whereas such an increase was less pronounced with the addition of BC. Moreover, a reduction in the relative abundance might have occurred in the second cycle as compared with the first cycle.

The proteobacteria were already abundant in the rhizospheric soil without the addition of biofertilizers and were further increased with the addition of doses of BO and BC in the two vegetative cycles, however the dose of 10% of BC in the second vegetative cycle reduced by half the abundance of this group, comparing them the control dose (Fig. 3). On the other hand, the phylum Firmicutes was in small abundance in the treatment of control rhizospheric soil, with greater colonization of these communities with the addition of doses of BO and BC in the two vegetative cycles.

Based on pooled DNA samples, estimates of species richness (Chao and ACE) and alpha diversity (Shannon and Inverse Simpson) suggest that the rhizospheric soil microbiome is both richer and more diverse than the microbial communities found in the pure biofertilizers. Among the biofertilizer treatments, BO showed higher richness and diversity estimates compared to BC. Consequently, rhizospheric soil treated with BO exhibited greater microbial richness and diversity than that treated with BC. In terms of vegetative cycle, the first cycle showed higher estimated richness and diversity than the second.

Due to the absence of biological replication in the sequencing analyses, all diversity metrics presented here should be interpreted solely as qualitative estimates. These findings provide preliminary insights into the potential differences in microbial community composition among treatments and over time but do not support statistical inference.

PCoA also suggested a tendency for separation between samples collected during the first and second vegetative cycles of *C. ciliaris*, indicating shifts in microbial community structure (Fig. S4). Additionally, the two pure biofertilizers exhibited relatively similar profiles in terms of beta diversity.

The analysis of the Theta-YC dissimilarity calculation using a distance matrix evaluated the association between treatments. In Figure S5, it can be seen that there was no well-defined grouping between the biofertilizers and their doses tested in the rhizospheric soil, but a discrete grouping of the samples was observed according to the vegetative cycle and doses, demonstrating the difference in the structure of the bacterial communities, the 1 st cycle in the lower doses (< 5.0%) and the 2nd cycle in the doses > 5.0%.

Diversity indices and ordination analyses were performed only for exploratory purposes using pooled samples and are presented in the Supplementary Material.

### Prediction of the functional content of the metabarcoding carried out by the 16 S rRNA gene

**Fig. 4:**
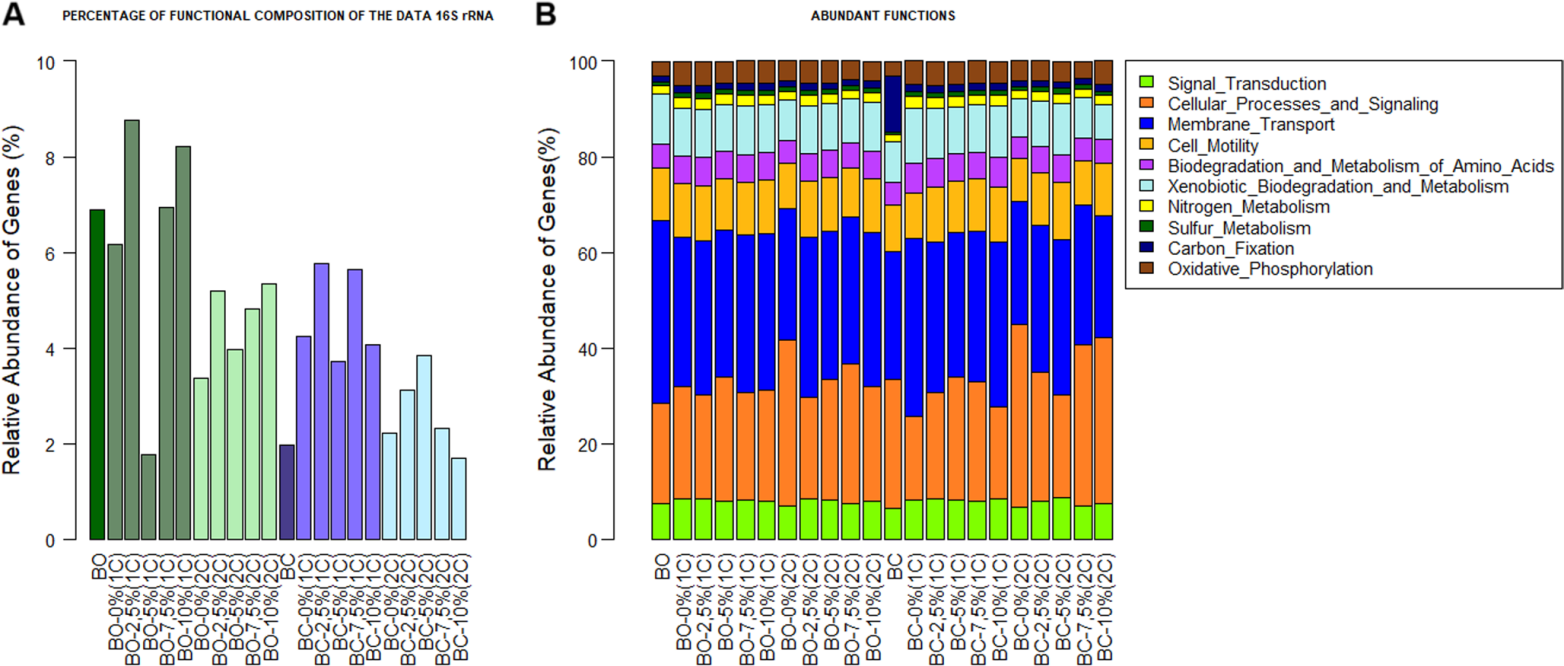
Percentage of the predictive functional profile of the microbial communities of the biofertilizers from sheep (BO) and goat (BC) manure and rhizospheric soils treated with increasing doses of such biofertilizers (0, 2.5, 7.5, and 10% of the soil volume) in two vegetative cycles of *C. ciliaris* (1 C: first cycle; 2 C: second cycle)

The functional composition of bacterial communities was more abundant in BO than in BC. The application of biofertilizers was associated with changes in the predicted functional gene profile of functional genes in the rhizospheric soil, mainly in 2.5 and 10% BO in both of the vegetative cycles, and in 2.5 and 7.5% BC in the first cycle (Fig. 4A).

The most abundant genetic functions in the samples were related to ABC membrane transport, functions related to the processing of genetic information, signal transduction, cellular processes and signaling, cellular motility, nucleotide metabolism, oxidative phosphorylation, enzymatic functions (peptidases), metabolism of amino acids, carbon fixation, carbohydrate metabolism, cofactor and vitamin metabolism, methane and lipid metabolism, biodegradation, and xenobiotic metabolism (Fig. 4B). It was also observed that the relative abundance of genes related to functionality might present a slight change from the first to the second vegetative cycle, e.g., genes related to amino acid metabolism, oxidative phosphorylation, and nitrogen metabolism, and bacterial communication were slight decreased with the addition of doses of biofertilizers.

## Discussion

The anaerobic biodigestion process, lasting 70 days, reduced by more than 90% the number of CFUs of total coliforms in the biofertilizers studied, demonstrating that the use of this process, which consists of the fermentation of organic residues by anaerobic bacteria, can result in a biofertilizer with lower density of total coliforms, a desirable characteristic for its use as an organic fertilizer. However, the addition of biofertilizers increased these populations in the rhizospheric soil. This was observed with the use of swine manure, leading to an increase in the number of total and fecal coliforms [24] and the application of poultry droppings and cassava peel, which caused an increase in the total number of coliforms (3.1 × 10^5^ CFU g^− 1^) compared to soil that received inorganic fertilizers (1.7 × 10^5^ CFU g^− 1^) [25].

The increase in MBC is strongly related to the higher availability of organic substrates in the rhizospheric soil, which influences nutrient cycling and energy flow. Quantifying MBC is crucial for studying these processes, as well as evaluating the dynamics of soil organic matter and the microbiota’s sensitivity to changes in ecosystems. Soil microorganisms, such as bacteria and fungi, play key roles in this context, with a particular focus on lactic acid bacteria, which aid in the biological control of plant diseases [26], and arbuscular mycorrhizal fungi (AMF), which promote plant growth and are involved in important soil ecosystem functions [5]. The highest doses of biofertilizers used in the present study increased rhizospheric soil organic matter by more than 500%, and this incorporation may have promoted an increase in MBC, thereby providing an environment favorable to the development of soil microbiota.

The presence of these microorganisms is influenced by agricultural practices, with organic systems proving more effective than conventional ones. Biofertilizers increased fungal populations in the rhizospheric soil. Other sources of organic matter (i.e. vegetal residue) can also increase fungal community in the soil. However, other studies show that organic fertilization tends to decrease or not alter the soil fungal community. These results may be related to organic fertilizer sources and their chemical characteristics [27], explaining the variation in the results obtained with the doses and biofertilizers tested in our study.

The reduction in glomerospores and mycorrhizal colonization observed with increasing biofertilizer doses may be associated with changes in nutrient availability and microbial interactions in the rhizosphere. Increased nutrient availability under fertilization regimes can reduce plant dependence on arbuscular mycorrhizal fungi, leading to lower levels of root colonization and sporulation. In addition, the incorporation of organic amendments may stimulate microbial activity and modify microbial interactions in the soil, influencing the establishment and reproduction of AMF [5, 28, 29]. Similar levels of mycorrhizal colonization in *C. ciliaris* under semi-arid conditions have been reported previously, indicating that environmental conditions and soil management can strongly influence AMF dynamics [6].

On the other hand, other sources of organic matter can increase sporulation such as that provided from plants (gliricidia) and animals (goat manure) due to the increase of organic matter in the soil [30]. These results confirm the importance of characterizing the organic sources used in the fertilization since the effects on the soil microbiota can vary according to the characteristics of the applied material.

On the other hand, improper management of animal waste can lead to contamination of soil and water, as many pathogenic microorganisms, such as *Salmonella* spp. and *Escherichia coli*, are excreted in animal manure and can proliferate in the environment [31, 32]. The persistence of *Salmonella* spp. in the soil corroborates other studies, since Arthurson [14] showed that *S. enterica* Enterica serovar Weltevreden strain 2007-60-3289-1 persists in the soil and spreads to the roots and leaves of spinach, without significant reductions, during a four-week evaluation period.

Additionally, the use of animal waste to produce biofertilizers can select for resistant bacteria, such as those from the *Clostridium* genus, which form spores and persist in the soil, especially when waste is treated anaerobically [33]. These factors highlight the importance of monitoring the microbial community in the soil, as counting and analyzing the microorganisms present helps in understanding soil fertility and assessing the impacts of agricultural practices on microbial balance and nutrient transformation processes.

Even though they are cultivable bacteria, no clostridium was isolated in this study, possibly because they require strict anaerobic conditions, which makes it difficult to isolate these microorganisms. In addition, the level of colonization by other bacterial species can inhibit their growth [34]. However, its presence was confirmed by the molecular technique.

The characterization of the bacterial microbiota by second-generation sequencing (NGS) showed that the biofertilizers revealed differences in the abundance of OTUs, which may be related to the digestive physiology of each animal species with short retention time in goats. The analyzes were performed on the Mothur, robust and integrated tool for OTU-based analyses that has been widely validated for microbial ecology. It is efficient for 16 S rRNA data and compatible with the objectives and available resources of this study. However, it is important to note that ASV-based pipelines, such as QIIME2 and DADA2 (https://qiime2.org/), offer greater taxonomic resolution and accuracy but require more computational resources.

Differences in the abundance of OTUS in biofertilizers is supported by findings from previous studies, which show that goat species present greater efficiency in the digestibility of the diet than sheep species do, eliminating lesser amounts of dry matter in their manure. This is a fact that might explain the reasons underlying a higher OM content and abundance and functionality of the bacterial microbiota in the rhizospheric soil treated with BO as compared with those in the rhizospheric soil treated with BC [35].

Furthermore, we can observe low levels of richness and diversity found in biofertilizers, suggesting that many microorganisms are eliminated in the process of biodigestion due to anaerobiosis. Biofertilizers showed less OTU richness and abundance than soil samples treated with increasing doses of them, since the microbial richness in soils exceeds that of other environments and is higher by orders of magnitude than biodiversity of plants and animals, for example [36].

Likewise, the concentration and amount of organic matter supplied by biofertilizers may have favored the survival and multiplication of bacteria in the soil [37], suggesting that doses of biofertilizers increase bacterial populations, with emphasis on the BO that increased the OM content in the soil and consequently favored the richness and abundance of species in this environment.

The most abundant phyla in the pure biofertilizers were Proteobacteria and Firmicutes, corroborating with studies that evaluated the structures and compositions of the microbial community in the composting processes of sewage sludge and cattle manure [38]. These phyla present bacterial communities predominant in organic waste composting processes, known as anaerobic or microaerophilic, which are favored in the biodigestion process [39].

Soil microbiome studies that show a stable bacterial community structure, have Proteobacteria, Acidobacteria, Actinobacteria, Verrucomicrobia, and Firmicutes as their most abundant phyla [40]. Thus, our findings are consistent with those of these authors.

The phyla Acidobacteria and Verrucomicrobia are involved in the degradation of biomass and availability of carbon. According to Lacerda Júnior et al. [41], acid bacteria have great metabolic potential for the production of extracellular enzymes involved in the degradation and use of hemicellulose, cellulose, and chitin. Similarly, Actinobacteria are efficient in degrading organic matter, with great importance in the decomposition of lignocellulose and cellulose hydrolysis [42], and they contribute a lot to the process of recycling carbon in the soil [43], whereas, Proteobacteria and Firmicutes promote plant growth, and having the capacity to convert nutritionally important elements from unavailable to available forms using biological processes, thus facilitating nutrient assimilation (NPK) by plants [43].

Therefore, the increase of these bacterial groups to the rhizospheric soil is quite interesting, since they grow in a variety of simple compounds, being associated with organic matter of the soil or the rhizosphere of the plant, playing an important role in the mineralization of organic matter, denitrification and fixation of nitrogen, degradation of proteins and carbohydrates, solubilization of phosphates in the soil, and also stand out as biological agents against plant pathogens, which can serve as potential biofertilizers [44].

It is also important to note that *C. ciliaris* root exudates can influence the diversity of the rhizospheric soil microbiome. The study by Jara-Servin et al. [45]. found a total of 24 phyla in the *C. ciliaris* microbiome, with a predominance of Actinobacteria, Proteobacteria and Acidobacteria and showed that *C. ciliaris* recruits microorganisms capable of thriving in allelochemical conditions and may be able to metabolize them. They also found that the composition of the microbial community changes depending on the stage of development of the *C. ciliaris*.

It is well known that biofertilizers tend to increase bacterial groups that perform important functions in the rhizospheric soil and that they are responsible for performing many critical functions of the ecosystem, turning this biodiversity an important biotic indicator of soil health [46]. However, the slight reduction in the abundance of OTUs and MBC from the first to the second vegetative cycle may be related to the excess of OM added to the soil with the use of biofertilizers, which causes an intense microbial activity due to the abundance of easily decomposable compounds. After this stage, the activity may decrease due to the mineralization of most of the organic compounds, in addition to a competitive interaction between the roots of the plants and the edaphic microorganisms [28]. Therefore, an alternative would be to alternate the use of biofertilizers with organic fertilization between the vegetative cycles of the grass, since this decrease may reflect in the reduction of soil functionality and consequently in the amount of forage produced, as found by da Silva et al. [17], who observed a reduction in the biomass of *C. ciliaris* from the first to the second vegetative cycle using BO.

In the predictive analysis of the functional content of biofertilizers and rhizospheric soils, it was also observed that the type of biofertilizer, the doses, and the vegetative cycles differentiate the provision of functions in the rhizospheric soil. Attention should be paid to the adoption of beneficial handling strategies, to avoid the loss of diversity and, consequently, the functional capacity of the rhizospheric soil, for reducing damages to the environment.

Research shows that introducing organic fertilizers to the soil results in changes in soil quality and functionality, providing substrates that significantly increase microbial biomass carbon activity and enzymatic activities. However, the function, structure of soil population germs were affected in long-term fertilization experiments [29].

Xue et al. [47], evaluated the potential functions of the soil microbial communities in conventional management systems with chemical inputs, systems with low chemical inputs, and organically managed systems. They demonstrated that the diversity of functional genes did not differ between low input and organic systems. Moreover, the abundance of genes involved in carbon, nitrogen, phosphorus, and sulfur cycles was higher in these systems as compared with that in the conventional system. Thus, the organic system, involving the use of biofertilizers, can help to support the functions mediated by microorganisms, particularly the most abundant ones found in the present study, which are related to the metabolism of amino acids, carbohydrates, lipids, vitamins, and minerals, which are important for sustaining ecosystem functions, including the provision of nutrients for crop growth.

Abundant functions found in this study, such as cellular processes and cell signaling, signal transduction, and motility, show the bacterial adaptive behavior concerning the biofertilizers added to the soil, since the mechanism of cellular communication plays a central role in the physiology and development of groups of bacteria, through the ability to coordinate activities, regulating the expression of specialized genes in response to population density, important for the survival of populations. Cellular processes and signaling allow bacteria to detect environmental changes and adjust their growth strategies, such as activating stress responses or utilizing new nutrients. Signal transduction converts external cues, like nutrient availability from biofertilizers, into intracellular actions that enhance survival and growth. Together, these adaptive behaviors optimize bacterial efficiency in nutrient cycling and promote soil health, making biofertilizers more effective in supporting plant growth [48].

The predicted functional profiles obtained from the PICRUSt analysis revealed a predominance of metabolic pathways associated with nutrient transport, energy metabolism, and cellular signaling, including ABC transporters, amino acid metabolism, carbohydrate metabolism, and oxidative phosphorylation. These functional groups are commonly associated with microbial communities involved in nutrient cycling and organic matter decomposition in soils. The increase in these predicted pathways may be related to the higher availability of organic substrates and nutrients observed in the rhizospheric soil after biofertilizer application, particularly the increases in organic matter and macronutrients. In this context, microbial communities may have responded to the greater availability of resources by activating metabolic pathways related to nutrient uptake and energy production. However, the increase in soil electrical conductivity at higher biofertilizer doses may have imposed osmotic stress on microorganisms, which can negatively affect microbial biomass and activity, helping to explain the reduction in microbial biomass carbon observed in the second vegetative cycle.

Although the predictive functional analysis using PICRUSt contributed to the characterization of potential microbial functions, it is important to recognize methodological limitations. This approach is based on closed-reference OTU picking and assumes that phylogenetically related taxa share similar genomic content, which can lead to biases, particularly in diverse environmental samples containing taxa not represented in reference databases such as GreenGenes. Moreover, the predictions reflect theoretical functional potential rather than actual gene expression or metabolic activity [49]. While alternative methods such as shotgun metabarcoding or metatranscriptomics would provide more direct and comprehensive functional data, they were not applicable in the present study. Additionally, more advanced prediction tools such as PICRUSt2, which incorporates improved reference genomes and hidden-state prediction, could yield more reliable inferences and represent a valuable option for future research. Therefore, the functional results should be interpreted as exploratory and descriptive. Nonetheless, their consistency with cultivation-based findings reinforces the ecological relevance of the observed patterns.

Thus, the biofertilizers evaluated in this study show strong potential for enhancing agricultural productivity in semiarid environments, as the research employed a two-cycle, dose-response experimental design that enabled a more nuanced temporal and quantitative analysis of changes in microbial structure and function. This approach provided novel insights into the non-linear and cycle-dependent effects of biofertilizer type and dose, contributing to a deeper mechanistic understanding of biofertilizer–microbiome interactions.

Previous studies on manure-based additives or biofertilizers have generally focused on single application events or short-term observations and reported linear effects on soil chemistry and microbial communities. However, little attention has been given to how dose-dependent responses evolve over successive growing seasons, especially in semi-arid regions where soil microbial dynamics are limited by available resources. This study used a double-cycle dose-response design to reveal that the effects of biofertilizer origin (sheep and goat) and dose are not static, but rather, change over time. This produces nonlinear, cycle-dependent patterns in microbial diversity and mycorrhizal interactions. This temporal dimension provides new insights into the thresholds at which beneficial effects are maintained or lost. From a practical standpoint, this approach identifies an optimal dose of around 5% of the soil volume that sustains microbial functionality while preventing diversity loss. This offers guidelines for the sustainable use of biofertilizers in semi-arid agroecosystems.

These findings underscore the importance of tailoring organic fertilization strategies to optimize microbial function, nutrient cycling, and the health of the soil in agroecosystems of arid lands. In order for these findings to be validated and applicable in real agricultural contexts, it is essential to carry out field studies that will allow the effectiveness of biofertilizers to be assessed in real situations, taking into account variables such as climate, soil, agricultural management, as well as the interaction of biofertilizers with different crops and ecosystems.

## Conclusions

In conclusion, biofertilizers derived from goat and sheep manure affect the microbial diversity, functional composition, and organic matter content of rhizospheric soils. However, excessive application over two consecutive vegetative cycles may reduce microbial diversity and functional potential. Results show that parsimonious application of biofertilizers at 5% of the soil volume increases microbial activity without compromising soil diversity or functionality.

Future long-term, replicated field studies are essential to validate these findings and extend them to different crops and management conditions in semi-arid regions.

## Supplementary Information

Below is the link to the electronic supplementary material.

Supplementary Material 1 (DOCX 1.77 MB)

## Data Availability

The data underlying this article are available in the paper and its online supplementary material.
