# Supplementary material for: Impact of biofertilizers from goat and sheep manure on rhizospheric microbial community of Cenchrus ciliaris
Source: Braz J Microbiol. 2026 May 11;57(1):138. doi: 10.1007/s42770-026-01938-4 (PMC13161455; doi:10.1007/s42770-026-01938-4)
Supplement: Supplementary file 1 — Supplementary Material 1 (DOCX 1.77 MB) [file 42770_2026_1938_MOESM1_ESM.docx]

**Impact of biofertilizers from goat and sheep manure on** **rhizospheric microbial community of** *Cenchrus ciliaris*

**Jennifer Figueiredo da Silva Oliveira^1^** [**https://orcid.org/0000-0002-8907-9565**](https://orcid.org/0000-0002-8907-9565)**; Gisele Veneroni Gouveia^2^** [**https://orcid.org/0000-0003-1074-5711**](https://orcid.org/0000-0003-1074-5711)**; João José de Simoni Gouveia^2^** [**https://orcid.org/0000-0002-0438-094X**](https://orcid.org/0000-0002-0438-094X)**; Luciana Correia de Almeida Regitano^3^** [**https://orcid.org/0000-0001-9242-8351**](https://orcid.org/0000-0001-9242-8351)**; Wilson Malago-Jr^3^** [**https://orcid.org/0000-0002-5802-4078**](https://orcid.org/0000-0002-5802-4078)**; Danillo Sales Rosa^4^** [**https://orcid.org/0000-0002-0447-5041**](https://orcid.org/0000-0002-0447-5041)**; Mário Adriano Ávila Queiroz^2^** [**https://orcid.org/0000-0001-7677-5273**](https://orcid.org/0000-0001-7677-5273)**; Mateus Matiuzzi da Costa^2^*** [**https://orcid.org/0000-0002-9884-2112**](https://orcid.org/0000-0002-9884-2112)**; Adriana Mayumi Yano-Melo^2^** [**https://orcid.org/0000-0003-2637-7183**](https://orcid.org/0000-0003-2637-7183)

^1^Instituto Federal Baiano, Senhor do Bonfim, Bahia 48970-000, Brazil

^2^Universidade Federal Do Vale Do São Francisco, Campus Ciências Agrárias, Petrolina, Pernambuco 56300-990, Brazil

^3^Embrapa Pecuária Sudeste, São Carlos, São Paulo 13560-970, Brazil

^4^Universidade Federal Rural de Pernambuco, Recife, Pernambuco 52171-900, Brazil

*Corresponding author: email address: [mmatiuzzicosta@gmaail.com](mailto:mmatiuzzicosta@gmaail.com). Telephone number: +55 (87) 988229388

**Supplementary Material**

**Fig. S1** Effect of applying biofertilizers from ovine (BO) and caprine (BC) manure at different doses (0, 2.5, 5.0, 7.5, and 10% of soil volume) on OM content (a), EC (b), CEC (c), N (first cycle) (d), P (e), N (second cycle) (f), and K (g) of the soil in two vegetative cycles of buffelgrass


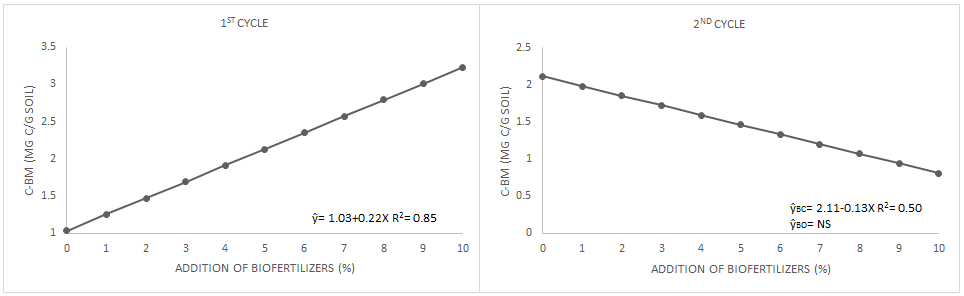


**Fig. S2.** The effect of biofertilizers on the MBC of the rhizosphere soil of *Cenchrus ciliaris* L., in the first (a) and second (b) vegetative cycles. Regression equation with normalized data with log (x).


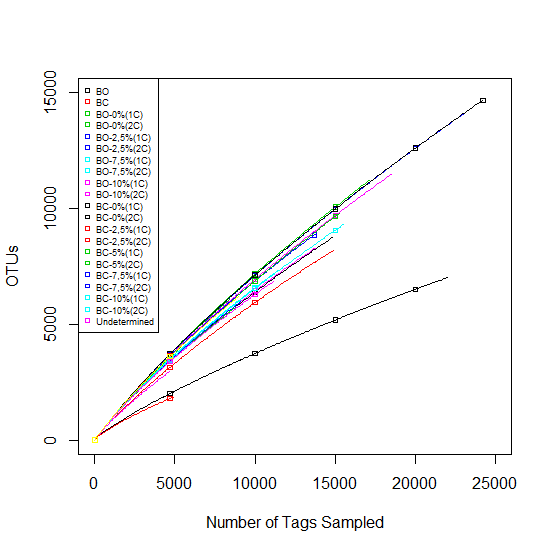


**Fig. S3.** Rarefaction curve showing the number of OTUs observed according to the number of sequences of the 16S rRNA gene analyzed from the biofertilizer (BO and BC) samples and soils treated with increasing doses of these biofertilizers (0, 2.5, 7.5, and 10% of the soil volume) in two vegetative cycles of buffelgrass (C1: first cycle; C2: second cycle).


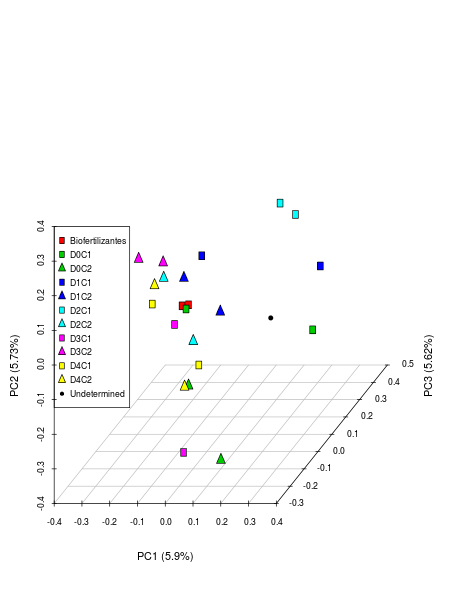


**Fig. S4.** Principal Coordinate Analysis (PCoA) of the beta diversity of the biofertilizer and soil samples treated in two vegetative cycles of buffel grass. This analysis was based on the grouping of Unweigted_UniFrac similarity matrices. Different geometric shapes represent each vegetative cycle of buffel-grass (C1 = first cycle and C2 = second cycle) and each color represents the dose of biofertilizer applied to the soil (Doses = D; D0, 1, 2, 3 and 4 = Dose 0%, 2.5%, 5%, 7.5% and 10% of the soil volume, respectively), with the exception of the red square and the black circle that represent biofertilizers and unclassified OTUs, respectively.


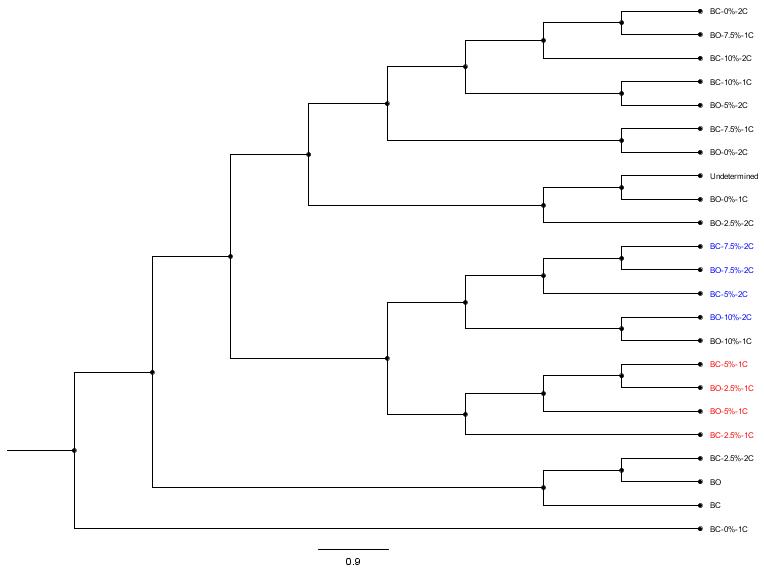


**Fig. S5.** Dendrogram from a cluster analysis based on a distance matrix, using Theta-YC dissimilarity calculation for the biofertilizer libraries (BO and BC) and soils treated with increasing doses of these biofertilizers (0, 2.5, 7.5, and 10% of the soil volume) in two vegetative cycles of buffelgrass (C1: first cycle; C2: second cycle). Grouping of the first and second cycle samples are shown in red and blue, respectively.

**Table S1.** Sequencing of the 16S rRNA gene amplicon libraries, including the number of raw sequences, trimmed sequences after quality filtering, read length, and the number of observed operational taxonomic units (OTUs).

| **Sample** | **Raw sequences** | **Trimmed sequences (n)** | **Read length (bp)** | **Observed OTUs** |
| --- | --- | --- | --- | --- |
| B1 | 44,623 | 27,327 | 240 | 1,87 |
| B2 | 16,007 | 7,501 | 240 | 1,675 |
| T1D0_1C | 37,14 | 20,089 | 240 | 3,44 |
| T1D0_2C | 19,661 | 9,828 | 240 | 3,411 |
| T1D1_1C | 50,028 | 27,327 | 240 | 3,414 |
| T1D1_2C | 29,569 | 16,697 | 240 | 3,36 |
| T1D2_1C | 9,541 | 5,022 | 240 | 3,25 |
| T1D2_2C | 23,206 | 13,682 | 240 | 3,247 |
| T1D3_1C | 39,484 | 22,511 | 240 | 3,359 |
| T1D3_2C | 27,581 | 16,083 | 240 | 3,187 |
| T1D4_1C | 48,325 | 27,906 | 240 | 3,431 |
| T1D4_2C | 31,979 | 18,554 | 240 | 3,16 |
| T2D0_1C | 44,209 | 23,681 | 240 | 2,907 |
| T2D0_2C | 14,326 | 7,023 | 240 | 3,352 |
| T2D1_1C | 35,751 | 19,215 | 240 | 3,312 |
| T2D1_2C | 17,948 | 10,432 | 240 | 3,145 |
| T2D2_1C | 22,88 | 11,474 | 240 | 3,333 |
| T2D2_2C | 19,713 | 11,106 | 240 | 3,177 |
| T2D3_1C | 31,453 | 18,344 | 240 | 3,212 |
| T2D3_2C | 13,865 | 7,095 | 240 | 3,148 |
| T2D4_1C | 23,537 | 13,896 | 240 | 3,137 |
| T2D4_2C | 10,028 | 5,379 | 240 | 2,756 |
